# Refining livestock mortality indicators: a systematic review

**DOI:** 10.12688/gatesopenres.13228.1

**Published:** 2021-04-19

**Authors:** Johanna T. Wong, Ciara Vance, Andrew Peters

**Affiliations:** 1 Supporting Evidence-Based Interventions - Livestock, University of Edinburgh, Easter Bush, Midlothian, EH25 9RG, UK

**Keywords:** Livestock mortality, mortality rate, mortality risk, perinatal mortality, neonatal mortality, young stock mortality.

## Abstract

**Background:** Livestock mortality impacts farmer livelihoods and household nutrition. Capturing trends in livestock mortality at localised or national levels is essential to planning, monitoring and evaluating interventions and programs aimed at decreasing mortality rates. However, livestock mortality data is disparate, and indicators used have not been standardised. This review aims to assess livestock mortality indicator definitions reported in literature, and define the ages where mortality has greatest impact.

**Methods:** A systematic review was conducted, limited to articles focussed on mortality of cattle, sheep and goats. Peer-reviewed articles in Web of Science until year 2020 were assessed for inclusion of age-based definitions for mortality indicators and data on age distribution of mortality. Indicator definitions for each species were collated and similar terms and age groups most targeted were compared. The cumulative distribution of age at mortality was compared across studies graphically where possible; otherwise, age patterns for mortality were collated.

**Results:** Most studies reported mortality risk rather than rate, and there was little agreement between indicator definitions used in the literature. The most common indicators reported were perinatal and neonatal mortality in cattle, and for perinatal, neonatal and pre-weaning mortality indicators for sheep and goats. Direct comparison of age distribution of mortality was only possible for cattle, which found that approximately 80% of all mortalities within the first 12 months had occurred by six months of age. A significant finding of the study is the variation in age groups for which mortality is reported, which impedes the comparison of mortality risk across studies, particularly for sheep and goats.

**Conclusions:** This study demonstrates the importance and value of standardising mortality risk indicators for general use, including a young stock mortality risk indicator measuring mortality in the highest risk period of birth to six months of age in cattle, sheep and goats.

## Introduction

Approximately 1 billion poor people globally are dependent on livestock for their livelihoods ( Ashley *et al*., 1999; FAO, 2009; Salmon *et al*., 2020). However, the benefits derived from livestock ownership, including income and household nutrition, are constrained by poor animal health and low productivity ( Perry *et al*., 2002). The impact of livestock disease has been cited in many publications e. g. Perry *et al*. (2013), but the ability to monitor change is limited as the available data is contained in disparate publications and reports, usually from individual countries, and there are few longitudinal studies of disease prevalence and impact. Donors to international development projects are increasingly interested in being able to monitor change in a country’s performance particularly in response to investment.

It has been recognised that there is a great disparity between the contribution that livestock make to agricultural GDP in many countries compared to relatively poor investment in development of the livestock sector ( Perry *et al*., 2002). In recent years, the Bill and Melinda Gates Foundation (BMGF) have been major investors in the low- and middle-income country (LMIC) livestock sector, and in particular, in animal health. However, it has become clear that development in LMIC agro-economies is hampered by the lack of data which can be used to prioritise policy investment decisions. Indeed, Mr. Bill Gates has himself stated *“Great science is helping to turn livestock into a pathway out of poverty for hundreds of millions of people…we have a lot of tools [such as] breeding, gene editing, vaccines…the lack of data makes us pretty uncertain about the right way to go’’* (W. Gates, Edinburgh, 2018). This constraint applies to national governments, non-governmental organisations (NGOs) and other donors.

In response to this, the University of Edinburgh’s Supporting Evidence-Based Interventions (SEBI) program, which is funded by the BMGF, aims to improve livestock data collection, curation and utilisation across the sector. This will enable the livestock community to make better investments and smallholder livestock keepers to make better-informed decisions, ultimately driving sustainable transformation of the livestock sector as a whole. Under the scope of this work, one of the key animal health indicators that SEBI will monitor on behalf of the BMGF is “livestock mortality rate”, currently defined as the total cumulative number of livestock deaths over the approximate average number of animals in the herd. The BMGF has set a target for SEBI to investigate interventions that can decrease livestock mortality by 10–15% over a 10-year period in their priority countries of Ethiopia, Nigeria and Tanzania. If such mortality reductions are achievable, this provides a significant opportunity to increase livestock productivity and in turn improve the livelihoods of smallholder farmers. To explore the feasibility of achieving this target, SEBI has been compiling evidence on current livestock mortality rates, causes, and possible interventions that may be able to achieve a significant mortality rate reduction. In addition, SEBI also aims to better define a set of indicators that the BMGF can use to monitor progress in their target areas of livestock health and productivity.

This review seeks to clarify the definition of “livestock mortality rate” in ruminants by first examining the rationale behind the use of mortality indicators, then exploring what definitions are currently used in the field, and in which age groups mortality has the greatest impact. The results of this review are then used to inform the selection of the best definition of mortality rate as an indicator to monitor development in animal health.

### Rationale behind use of mortality indicators

***Use in human health.*** Mortality rates are used extensively in human health literature to monitor progress in human development. However, mortality rates are usually qualified by factors such as age, life stage, or cause. To illustrate this, Table 1 shows the definitions of selected Sustainable Development Goal (SDG) targets and indicators measuring mortality rates from SDG 3: “Ensure healthy lives and promote well-being for all at all ages” ( UNSD, 2020a; UNSD, 2020b). The specificity of each mortality indicator allows comparison across time and space. Changes in trend can also be traced back to a relatively discrete number of factors for each indicator, and therefore drive targeted research, programs and interventions.

**Table 1.** Selected SDG indicators from SDG 3 that measure human mortality rates ( UNSD, 2020a; UNSD, 2020b).

Target	Indicator	Definition
**3.1:** By 2030, reduce the global maternal mortality ratio to less than 70 per 100,000 live births.	**3.1.1:** Maternal mortality ratio	The annual number of maternal deaths from any cause related to or aggravated by pregnancy or its management (excluding accidental or incidental causes) during pregnancy and childbirth or within 42 days of termination of pregnancy, irrespective of the duration and site of the pregnancy, per 100,000 live births, for a specified year.
**3.2:** By 2030, end preventable deaths of newborns and children under 5 years of age, with all countries aiming to reduce neonatal mortality to at least as low as 12 per 1,000 live births and under-5 mortality to at least as low as 25 per 1,000 live births.	**3.2.1:** Under-five mortality rate	The probability (expressed as a rate per 1,000 live births) of a child born in a specified year or period dying before reaching the age of five if subject to current age-specific mortality rates.
	**3.2.2:** Neonatal mortality rate	The probability that a child born in a specific year or period will die during the first 28 completed days of life if subject to current age-specific mortality rates, expressed per 1,000 live births. Neonatal deaths may be subdivided into early neonatal deaths (first seven days of life), and late neonatal deaths (after 7th day but before 28th completed day of life).
**3.4:** By 2030, reduce by one third premature mortality from non- communicable diseases through prevention and treatment and promote mental health and well-being.	**3.4.1:** Mortality rate attributed to cardiovascular disease, cancer, diabetes, or chronic respiratory disease	The percent of 30-year-old people who would die before their 70th birthday from cardiovascular diseases, cancer, diabetes, or chronic respiratory diseases, under the assumption that the experienced mortality rate does not change over time, excluding other causes of death such as accidents or HIV/AIDS. This indicator is calculated using the life table method.
**3.9:** By 2030, substantially reduce the number of deaths and illnesses from hazardous chemicals and air, water and soil pollution and contamination	**3.9.1:** Mortality rate attributed to household and ambient air pollution.	The mortality attributable to the joint effects of household and ambient air pollution and can be expressed as per 100,000 population for any given population group (e.g. children under 5 years of age).
	**3.9.2:** Mortality rate attributed to unsafe water, unsafe sanitation and lack of hygiene	The number of deaths from unsafe water, unsafe sanitation and lack of hygiene in a year per 100,000 population.
	**3.9.3:** Mortality rate attributed to unintentional poisoning	The number of deaths from unintentional poisonings in a year per 100,000 population.

SDG = Sustainable Development Goals; UNSD = United Nations Statistics Division; HIV = human immunodeficiency virus; AIDS = acquired immune deficiency syndrome.

Although only encompassing narrow age groups, neonatal, infant and under-five mortality rates are often used as barometers for overall population health ( UNSD, 2020a; WHO, 2021). Reidpath & Allotey (2003) examined the ability of infant mortality rate (IMR), defined as the number of deaths in children under 1 year of age per 1000 live births in the same year, to represent whole population health compared to the more comprehensive measure of disability-adjusted life expectancy (DALE), which accounts for mortality as well as non-fatal morbidity. The analysis found a strong, linear correlation between IMR and DALE, showing that IMR is a feasible and useful indicator of whole population health. Reidpath & Allotey (2003) also discussed the difference in resources required to collect data for each indicator, highlighting that the simpler IMR was more feasible to monitor in resource-poor countries.

This is important considering the resources that are required to collect a broad set of data to construct complex indicators, compared to the resource limitations often faced in LMICs.

***Use in livestock health.*** Livestock fulfil multiple roles in various parts of the world; however, their primary roles are generally for income generation, food, and employment ( Herrero *et al*., 2013; Salmon *et al*., 2020). This focus on production marks a significant difference in perspective between human and animal health and means that indicators that measure progress in humans may not be directly transferrable to livestock. In addition, the mixture of public and private interests in livestock production complicates and often limits the availability of public resources for animal health. Practically, this necessitates efficiency in resource allocation for data collection, analysis, and action.

In animal health, attempts to devise a system analogous to disability-adjusted life years (DALYs) or DALEs for humans has not, as yet, gained general acceptance in the livestock development community ( Shaw *et al*., 2017). Therefore, at the present time, SEBI is attempting to define an indicator of livestock mortality that is a stable reflection of the animal health status of a country.

Livestock mortality has always been an important issue, as loss of stock represents a loss of wealth, livelihood, nutrition, genetic material, and a waste of investment, whether financial or through labour. The magnitude of lost value experienced by livestock keepers may be comparatively greater for those whose livestock fulfil several purposes, as is the case in many LMICs.

A suite of mortality indicators available for use in animals, as described by Thrusfield & Christley (2018), are presented in Table 2. Issues with the current definitions are immediately evident: the authors do not define the age for which calf/lamb/kid or neonatal mortality rates apply, as “there is not a universal agreement on the age at which animals cease to be neonates in veterinary medicine”. With this range of indicators and a lack of consensus on age groupings, it is important to gain a better understanding of what the term “mortality rate” actually means in a practical sense, particularly when referencing specific age groups. To this end, a literature search was performed with the aim to collate and review the terms that are most frequently used within the livestock farmer, professional and scientific research communities, and to review the age categories which have the highest incidence of mortality with a view to refining the current definition of “livestock mortality rate”. This review is reported in line with the Preferred Reporting Items for Systematic Reviews and Meta-Analyses (PRISMA) ( Wong, 2021).

**Table 2.** Mortality indicators used in veterinary epidemiology ( Thrusfield & Christley, 2018).

Indicator	Numerator	Denominator
Cumulative mortality	Number of individuals that die during a particular period	Number of individuals in the population at the beginning of that period
Mortality rate or mortality density	Number of deaths due to a disease that occurs in a population during a particular period of time	The sum, over all individuals, of the length of time at risk of dying
Death rate or crude mortality rate ^a^	The total mortality rate for all diseases (rather than just one)	The sum, over all individuals, of the length of time at risk of dying
Case fatality	Number of deaths	Number of diseased animals
Crude death rate (in 10 ^b^ animals)	Number of deaths occurring	Average population
Age-specific death rate (in 10 ^b^ animals)	Number of deaths among animals in a specified age group	Average number in the specified age group
Calf/lamb/kid mortality rate (in 10 ^b^ animals)	Number of deaths under a specified age	Number of live births
Neonatal mortality rate (in 10 ^b^ animals)	Number of deaths under a specified age	Number of live births
Foetal death rate (in 10 ^b^ animals)	Number of foetal deaths	Number of live births plus foetal deaths
Cause-specific death rate (in 10 ^b^ animals)	Number of deaths from a specified cause	Average population

In these mortality indicators commonly used in livestock, there is no consensus on age definitions for “calf”, “lamb”, “kid”, or “neonatal”. ^a ^The distinction between mortality rate and death rate is not always clear when reported. ^b^ Usually a whole number between 2–6.

## Methods

### Eligibility criteria

A preliminary search was performed in Google to collect commonly used terminology from a mixture of academic and professional publications. This search showed that indicators used to monitor mortality rates are often specific for age groups (especially young animals), defined time periods (e.g., annual, a study duration), or specific diseases (i.e., case fatality rates). Common terms encountered during this preliminary search contributed to the development of a search strategy (outlined in Table 3) to retrieve articles reporting on mortality rates and age at mortality in cattle (both dairy and beef production systems), sheep, and goats.

**Table 3.** Initial Web of Science search terms and results.

Search stem	Additional search term	Number of results
Mortality rate AND	Cattle OR bovine OR calf OR sheep OR ovine OR lamb OR goat* or caprine OR kid	4305
Perinatal mortality AND	Livestock	17
	Cattle OR bovine OR dairy OR beef OR calf OR calv*	287
	Sheep OR ovine OR lamb*	252
	Goat* OR caprine OR kid OR kids	38
Neonatal mortality AND	Livestock	50
	Cattle OR bovine OR dairy OR beef OR calf OR calv*	667
	Sheep OR ovine OR lamb*	394
	Goat* OR caprine OR kid OR kids	129
(Pre-weaning OR preweaning OR pre wean* mortality) AND	Livestock	83
	Cattle OR bovine OR dairy OR beef OR calf OR calv*	1,490
	Sheep OR ovine OR lamb*	543
	Goat* OR caprine OR kid OR kids	207
Young stock mortality OR youngstock mortality AND	Livestock	15
	Cattle OR bovine OR dairy OR beef OR calf OR calv*	61
	Sheep OR ovine OR lamb*	18
	Goat* OR caprine OR kid OR kids	8
**Total**		**8564**

The large number of results for cattle contrasts with the limited amount of literature available for goats.

***Inclusion criteria.*** Articles from all countries published between 1900 and 2020 were considered for inclusion. A wide date range was used due to the scoping nature of the indicator definition review, and to maximise the inclusion of data from LMICs. The search included peer-reviewed journal articles (original research, secondary data analysis and reviews for both mortality indicator definitions and age distribution of mortality, and method articles also for indicator definitions). Data from published research reports related to the selected journal articles were included if they expanded on or clarified definitions used in the article. For age distribution of mortality, articles studying interventions were only included if baseline data were reported, and only baseline data was considered for inclusion. To ensure all nuances in indicator definitions were captured, articles were only included in the indicator definition section if they were in English.

***Exclusion criteria.*** For both studies, articles were excluded if their abstracts or full text papers were not available. For the indicator definition section, studies were excluded if the definitions were based on events such as ear-tagging or weaning rather than age group. For age distribution of mortality, articles that only reported on cause-specific mortality rates, or articles that presented experimental data in laboratory settings that did not reflect realities in the field were excluded.

An overview of the criteria used to identify literature relating to the two purposes of this review is presented in in Table 4.

**Table 4.** Study selection criteria.

Domain	Criteria	
	Definition of mortality rate	Age at mortality
Date range	1900–2020	1900–2020
Geographical scope	Global	Global
Type	Peer-reviewed journal article, including original research, secondary data analysis, method articles and reviews	Peer reviewed journal article, including original research, secondary data analysis and reviews.
Specific details	Defined or reported on mortality rates for explicit age groups, including those reporting on specific diseases. Articles that defined mortality based on events, such as ear-tagging or weaning, were excluded if the typical or average age at these events were not reported.	Reported incidence of all-cause mortality by age. Articles that reported mortality rates within study cohorts but did not aim specifically to monitor or explore mortality, or articles citing published mortality rates as part of study backgrounds were excluded.
Exclusions	Abstract unavailable Full text unavailable	Abstract unavailable Full text unavailable Cause-specific mortality rates Data from laboratory-based settings that are unlikely to be replicated in the field.
Language	English only	All languages as long as specific details on study design and age at mortality were able to be translated using Google Translate.

### Search

Literature searches were then performed in Web of Science (core databases) in June 2020. An initial search for “mortality” in combination with the full series of additional search terms yielded too broad a range of results, many of which were not related to livestock production. The search strings were refined, and the specific search strings used are presented in Table 3. A Google Scholar literature search for “livestock mortality rate” was also performed to ensure as many articles were included, as well as inclusion of relevant articles found within reference lists.

### Study selection

For all terms, article titles were reviewed for relevance to livestock mortality rates. Article abstracts for relevant titles were then reviewed by two authors (J. W. and A. P.) according to the selection criteria ( Table 4) and articles selected for full-text review. Full-text articles were reviewed by J. W. and A. P., and disagreements on inclusion were resolved through discussion. Where multiple papers arose from the same research study, or where research groups used the same definition or presented the same information across multiple papers, only the most detailed publication was included.

### Data extraction

Data were extracted into a piloted form in Microsoft Excel version 2102. Data extracted included: author/s, citation, country of origin and income group, species and breed, production system characteristics, whether the aim/objectives of study directly related to livestock mortality, study type, recruitment procedure, sample size, time span covered, mortality indicator name, indicator enumerator, indicator denominator, whether abortions, stillbirths or culling events were included, and age distribution of mortality events recorded.

### Data analysis

For the study of mortality indicator definitions, definitions were grouped by species and age range, and studied for patterns or common age ranges to produce a narrative summary. Given the scoping nature of this section of the review, studies were not individually assessed for bias.

For the study on age distribution of mortality, studies were first grouped into age ranges studied. Where studies overlapped in age range coverage, mortality risk by calendar month (365.25 days/12) was calculated, and the cumulative mortality risk by age in months graphed for each study along with the average mortality risk and standard deviation across studies. These studies were assessed for bias using the Risk of Bias Tool for Prevalence Studies ( Hoy *et al.,* 2012). Where studies did not have commonalities in age range, the results were included in a narrative summary. These studies were not assessed for bias. All analysis was done in Excel version 2102.

This review was not registered in PROSPERO and did not require ethical approval.

## Results

**Figure 1.**
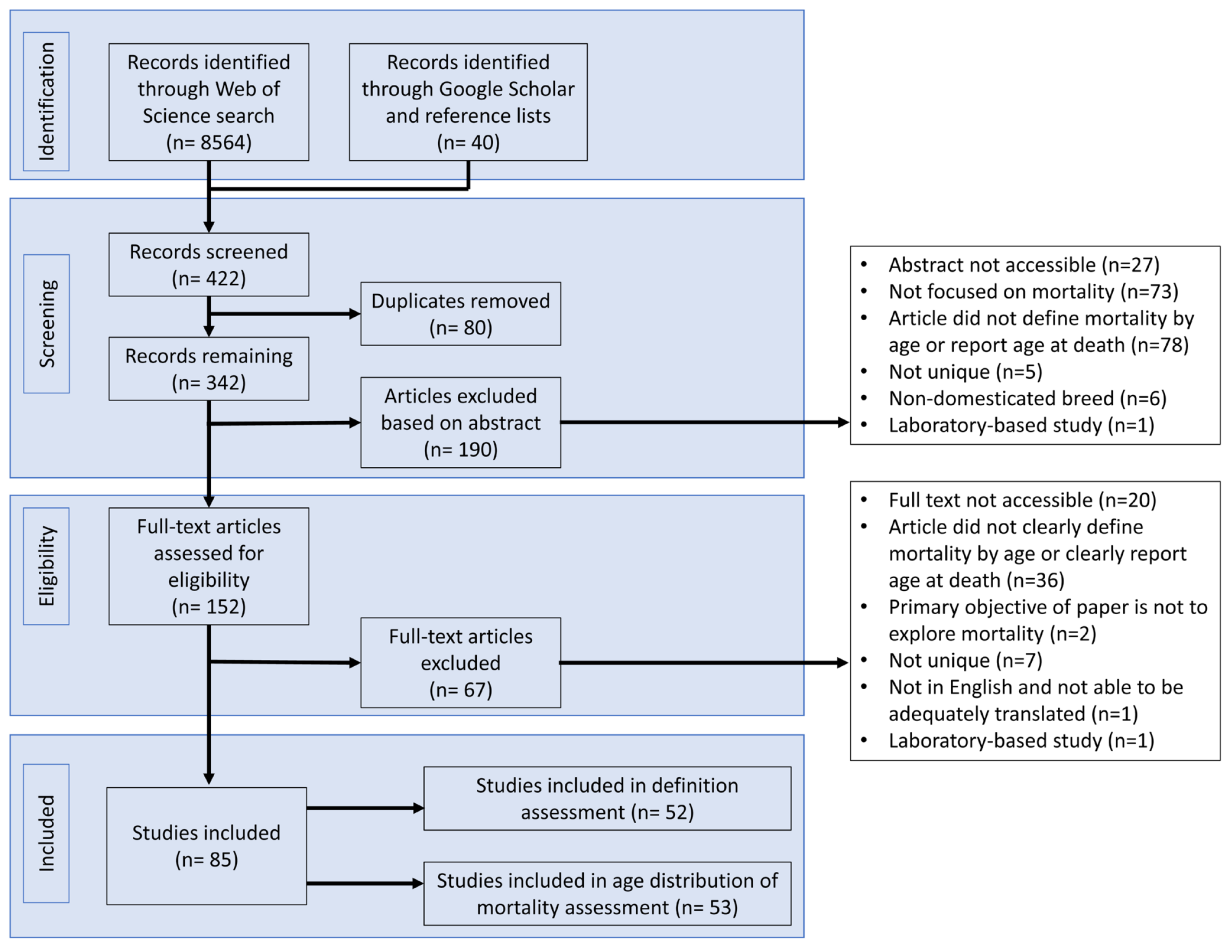
PRISMA flow chart for article screening and inclusion (adapted from Moher *et al.*, 2009). Of the 8604 articles identified by the literature search, 422 titles were assessed as relevant. Eighty duplicates were removed, before 190 articles were excluded based on their abstract contents. Sixty-seven articles were excluded on assessing the full text, leaving 85 articles for inclusion into the study.

To give a general overview of the popularity of each term and the availability of literature for each species, the number of returns for each search is included in Table 3. In terms of species distribution of literature, it is evident that cattle account for the largest proportion of articles, followed by sheep, with goats having very poor representation. Results of the literature search are presented in Figure 1. The database search in Web of Science yielded 382 potentially relevant articles based on title, while 40 articles were identified through Google Scholar and article reference lists. Duplicates (n=80) were removed, before 190 articles were excluded based on their abstracts. Of the 152 full-text articles assessed for eligibility, 85 articles were included in the review. As some articles both defined mortality rate based on age and reported on age distribution of mortality, 52 articles were included in the study on mortality rate indicator definitions, while 53 articles were included for the age distribution of mortality study.

### Defining “mortality rate”

The literature search for “mortality rate” in cattle, sheep and goats mostly yielded articles focussed on mortality in calves, lambs and kids. Few articles defined specific age groups for older animals – general herd or flock mortality rates tended to be reported instead. For all species and age groups, the literature was divided into those studies reporting a true mortality rate, and those reporting mortality risk.

***Mortality incidence rate versus mortality incidence risk.*** The Centres for Disease Control and Prevention (CDC) define mortality rate as a “measure of the frequency of occurrence of death in a defined population during a specified interval” ( CDC, 2012). For livestock, movement of animals in and out of the herd, from mortality, sale, or slaughter, is common and makes quantifying the “population” as a denominator in the ratio, more complex than in static populations. The most accurate way to define a varying population is to calculate the number of animal-days-at-risk, thereby no longer counting animals as being at risk from the day they leave the population ( Thrusfield & Christley, 2018). Mortality incidence rate is often expressed per 100-animal-months or -years. However, crude mortality rates, more correctly known as mortality risk, are often used. These use estimates of the population, such as the number of animals counted or average herd/flock sizes, as a denominator and are accepted proxies. Mortality risk is commonly reported as “mortality rate” ( Thrusfield & Christley, 2018). Within the 52 included studies, 42 (81%) reported mortality risk, four reported mortality rate (8%), four studies reported both (8%) and in two review articles, it was not always clear whether the included articles reported risk or rate.

***Age ranges used to define mortality rate.*** For cattle, the literature search identified 30 articles that used 20 mortality indicators ( Table 5). Geographically, the 30 articles spanned 18 countries, with two studies having a global coverage. Using World Bank Group classifications ( World Bank Data Team, 2019), nine were from high-income countries (HICs), and nine were from LMICs.

Perinatal and neonatal mortality indicators were the most commonly reported. For perinatal mortality, though there was variation between authors on definitions, most included stillbirths and measured mortality between birth and up to 24 or 48 hours, while only a small number included abortions. There was similar variation in definitions for neonatal mortality, although from birth or 2–3 days through to one month of age was common. For older calves, there was a complete lack of consensus as to what age range indicators included.

**Table 5.** Definitions for cattle mortality indicators reported in peer-reviewed literature.

Indicator	Definition/Rationale	Country	Income level ^a^	System (if available)	Reference
Perinatal calf mortality	Mortalities from birth to 12 hours	The USA	HIC	Beef	Wittum *et al*., 1994
	Stillborn or death within 24 hours after birth	Switzerland	HIC	Dairy and beef, mixture of systems	Bleul, 2011
	Calf born dead or dying within 24 hours of parturition/Number of calves born	The UK	HIC	Dairy	Brickell *et al*., 2009
	Calves born dead and those that died within 24 hours/Total number of calves born	Germany	HIC	Dairy	Gundelach *et al*., 2009
	Death within 24 hours of calving	Ireland	HIC	Dairy	Mee *et al*., 2008
	Death of a full-term calf either during parturition or up to 48 hours	Global	-	Dairy, mixed systems	Cuttance & Laven 2019
	Abortions, stillbirths and deaths to 48 hours/Total number of calves born	Slovenia	HIC	Dairy	Voljc *et al*., 2019
	Mortalities in first 48 hours post-partum/Number of calvings	Switzerland	HIC	Extensive beef	Busato *et al*., 1997
	Death of live-born animals within 48 hours of life	Ethiopia	LMIC	Mixed crop-livestock, pastoral/ agropastoral, & market-oriented urban/peri-urban dairy production	Fentie *et al*., 2020
	Mortalities from 0–48 hours	Switzerland	HIC	Dairy	Mock *et al*., 2020
	Mortalities from 0–2 days of age/overall number of births, *and* Mortalities from 0–2 days of age /number of calf days.	France	HIC	Dairy	Raboisson *et al*., 2013
Newborn calf mortality	Number of calves born dead or died between 0–6 days/Total number of calves born	Finland	HIC	Large-scale dairy farms with free-stall barns and either parlour milking or automated milking	Seppa-Lassila *et al*., 2016
Neonatal mortality	Abortions recorded after mid-gestation, stillbirths, and deaths within 48 hours	The USA	HIC	Dairy	Dhakal *et al*., 2013
	Mortalities from birth to 7 days of age	The USA	HIC	Beef (Research centre)	Azzam *et al*., 1993
	Mortalities from birth to 28 days of age/Number of liveborn calves	Bangladesh	LMIC	Intensive dairy (breeding station)	Debnath *et al*., 1995
	Mortalities from birth to 28 days of age/ Number of live births monitored during the neonatal period of the calving season of interest.	Turkey	LMIC	Dairy	Erdogan *et al*., 2009
	Mortalities from 0 to 28 days of age	Global	-	Beef or dairy	Uetake, 2013
	Mortalities from 12 hours to 45 days of age	The USA	HIC	Beef	Wittum *et al*., 1994
	Mortality between 1 – 30 days of age over total number of calves in this age group.	Denmark	HIC	Unspecified	Fuerst-Waltl & Sorenson, 2010
	Death of newborn animals between 48 hours and 1 month of age	Ethiopia	LMIC	Mixed crop-livestock, pastoral/ agropastoral, & market-oriented urban/peri-urban dairy production	Fentie *et al*., 2020
	Mortalities from 2 days to 30 days of age/Total number of calves born	Slovenia	HIC	Dairy	Voljc *et al*., 2019
	Mortalities from 3 days to 1 month of age/overall number of births, *and* Mortalities from 3 days to 1 month of age/number of calf months.	France	HIC	Dairy	Raboisson *et al*., 2013
Postnatal calf mortality	Death of a calf from birth to 14 days inclusive/Calf-days-at-risk	The Netherlands	HIC	Dairy or beef	Santman-Berends *et al*., 2019
Early post-natal mortality	Mortality between 1–21 days of age/Total number of calves born alive	Mexico	LMIC	Dairy	Mellado *et al*., 2014
Pre-weaning calf mortality	Mortality of calves up to weaning at 8 weeks of age/Total number of calves eligible for study	The UK	HIC	Intensive dairy production	Mahendran *et al*., 2017
	Mortalities from 15 to 55 days of age/Total number of ear- tagged calves, *and* Mortalities from 15 to 55 days of age/Calf- days-at-risk	The Netherlands	HIC	Dairy or beef	Santman-Berends *et al*., 2019
Early pre-weaning mortality	Mortality of young stock between 1–3 months of age	Ethiopia	LMIC	Mixed crop-livestock, pastoral/ agropastoral, & market-oriented urban/peri-urban dairy production	Fentie *et al*., 2020
Young calf mortality	Number of deaths in calves aged 21 to 90 days/Number of cow- days in the farm unit belonging to the respective age group	Estonia	HIC	Dairy farms, systems not specified	Reimus *et al*., 2020
Calf mortality	Mortality in unweaned calves 1–5 months of age ^b^/Animal-days- at-risk	Estonia	HIC	Mainly extensive beef production	Motus *et al*., 2018
Calf mortality	Mortality of a bovine animal born alive and registered dead before 6 months of age/ The total number of calf-time (days) at risk.	The UK	HIC	Not specified	Ortiz-Pelaez *et al*., 2008
Rearing period mortality	Mortalities from one to six months of age/overall number of births, *and* Mortalities from one to six months of age/number of calf months.	France	HIC	Dairy	Raboisson *et al*., 2013
Calf mortality	Number of calves dying between 7 and 180 days/Number of live calves at 7 days of age	Finland	HIC	Large-scale dairy farms with free-stall barns and either parlour milking or automated milking	Seppa-Lassila *et al*., 2016
Calf mortality	Mortality between 31 – 180 days of age over total number of calves in this age group.	Denmark	HIC	Not specified	Fuerst-Waltl & Sorenson, 2010
Post-weaning mortality	Mortality between 8 weeks and 14 months of age/Total number of eligible calves less calves that died pre-weaning	The UK	HIC	Intensive dairy production	Mahendran *et al*., 2017
Weaned calf mortality rate	Mortalities from 56 days to one year of age/Calf-days-at-risk	The Netherlands	HIC	Dairy or beef	Santman-Berends *et al*., 2019
Young stock mortality	Mortalities from birth to weaning age (up to one year for cattle)/ Number of live births within that particular study year	Ethiopia	LMIC	Mixed crop-livestock, pastoral/ agropastoral, & market-oriented urban/peri-urban dairy production	Fentie *et al*., 2016
Calf mortality/calf mortality rate	Number of mortalities in the first year of life in liveborn calves/ total number of calf-months in the study	Kenya	LMIC	Smallholder dairy	Gitau *et al*., 1994
Cumulative calf mortality	Number of calf mortalities from birth to one year	Cote d’Ivoire	LMIC	Traditional systems	Knopf *et al*., 2004
Young stock mortality	Number of deaths in stock less than one year of age/ Animal days at risk. Mortality risk was calculated from mortality rate	Tanzania	LMIC	Smallholder dairy cattle	Swai *et al*., 2010
Calf mortality/calf mortality rate	Death within first year of life, not including abortions	Mali	LMIC	Peri-urban traditional, modern and station management systems	Wymann *et al*., 2006
Late pre-weaning mortality	Mortality of young stock between 3-12 months of age	Ethiopia	LMIC	Mixed crop-livestock, pastoral/ agropastoral, & market-oriented urban/peri-urban dairy production	Fentie *et al*., 2020
Young animal mortality	Number of calves <18 months that died during the observation period/ number of young animals born in the same period	Afghanistan	LMIC	Village production systems	Schreuder *et al*., 1996
Youngstock mortality 6-19 months	Mortalities from 6-19 months of age/Animal-days-at-risk	Estonia	HIC	Mainly extensive beef production	Motus *et al*., 2018
Youngstock mortality	Number of all-cause mortalities in stock < 3 years of age/Animal years-at-risk	Tanzania	LMIC	Smallholder farms	Swai *et al*., 2009
Annual adult mortality	Number of adult (>18 months old or mated) cattle that died (including emergency slaughter) during a one-year observation period/ number of adult animals present at the start of the year	Afghanistan	LMIC	Village production systems	Schreuder *et al*., 1996
Youngstock mortality from 20 months	Mortalities in cattle older than 20 months of age/Animal-days- at-risk	Estonia	HIC	Mainly extensive beef production	Motus *et al*., 2018

^a^ As classified by The World Bank Group: LMIC = low- and middle-income country; HIC = high-income country. ^b^ The authors based this definition on their observation that the first month of life is a high-risk period for calf mortality

For sheep, 20 studies were identified, reporting on 16 indicators of mortality ( Table 6). These articles originated from 10 countries, including two HICs and eight LMICs and one article having a global scope. Perinatal, neonatal and pre-weaning mortality rates were most commonly reported. The definition of perinatal mortality varied greatly between authors, with indicators covering death between birth to 24 hours, 48 hours, seven days, and 14 days. There was a greater level of consensus for definitions of neonatal mortality, with most authors including mortalities from birth to 28 or 39 days of age. Pre-weaning mortality was most reported to be between birth to 90 days.

**Table 6.** Definitions for sheep mortality indicators reported in peer-reviewed literature.

Indicator	Definition/Rationale	Country	Income level ^a^	System (if available)	Reference
Perinatal mortality	Total losses caused by abortions, stillbirths, and neonatal mortality (to 14 days of age) in one breeding season/Sum of aborted, stillborn and lambs born alive	Jordan	LMIC	Extensively managed	Aldomy *et al*., 2009
	Mortalities within 24 hours of birth/Total number of lambs born	The UK	HIC	Multiple	Binns *et al*., 2002
	Stillbirths and lambs that died on their first day of life	Germany	HIC	Conservation grazing and non- seasonal production system	Voigt *et al*., 2019
	Mortalities within 48 hours of birth	Jordan	LMIC	Transhumant and sedentary systems	Al-Khaza'leh *et al*., 2020
	Death of live-born animals within 48 hours of life	Ethiopia	LMIC	Mixed crop-livestock, pastoral/ agropastoral, & market-oriented urban/peri-urban dairy production	Fentie *et al*., 2016
	Death up to seven days of age	Norway	HIC	Multiple	Holmoy & Waage, 2015
	Death within 15 days of birth/Total number of lambs born	India	LMIC	Semi-intensive and intensive systems on a research station	Mandal *et al*., 2007
Early neonatal mortality	Liveborn lambs that died between 0-5 days of life, including stillbirths/Total number of liveborn lambs	Norway	HIC	Multiple	Holmoy & Waage, 2015
Neonatal mortality	Mortalities between 0-5 days of age	Norway	HIC	Multiple	Holmoy *et al*., 2012
	Death of lambs during the first week of life	The UK	HIC	Multiple	Gascoigne *et al*., 2017
	Number of mortalities between birth and 14 days of age/Total number of lambs born alive	Jordan	LMIC	Extensively managed flocks	Aldomy *et al*., 2009
	The number of lambs dying during the first 28 days of life/The total number of lambs born alive	N/A	N/A	N/A	Fragkou *et al*., 2010
	Mortalities during the first four weeks of life	Turkey	LMIC		Gokce *et al*., 2013
	Mortalities during the first 28 days of life	India	LMIC		Gowane *et al*., 2018
	Mortalities from birth up to 30 days of age	Ethiopia	LMIC	On-station highland sheep	Bekele, *et al*., 1992
	Mortalities between 48 hours and 1 month after birth	Jordan	LMIC	Transhumant and sedentary systems	Al-Khaza'leh *et al*., 2020
	Deaths of newborn animals between 48 hours and 1 month of age	Ethiopia	LMIC	Mixed crop-livestock, pastoral/ agropastoral, & market-oriented urban/peri-urban dairy production	Fentie *et al*., 2016
Late neonatal mortality	Liveborn lambs that died between 6-14 days after birth/Total number of lambs alive at day 6 postpartum	Norway	HIC	Multiple	Holmoy & Waage, 2015
Pre-weaning mortality rate	Mortality from birth to 60 days of age/Number of lambs born alive	Jordan	LMIC	Extensive and semi-extensive production	Abdelqader *et al*., 2017
	Number of lambs born dead or alive but dying within 60 days post-lambing	Mexico	LMIC	Intensively reared lambs	Mellado *et al*., 2016
	Number of deaths until 90 days of age/Total births	Ethiopia	LMIC	Smallholder farms using tethered feeding or pastoralist systems	Deribe *et al*., 2014
	Death between 0-3 months of age/Total number of lambs born	India	LMIC	Semi-intensive and intensive systems on a research station	Mandal *et al*., 2007
	Number of lamb mortalities between 0 – 90 days of age/Total number of lambs born	Egypt	LMIC	Barki sheep raised on a research station	Sallam, 2019
	Mortality between 1-3 months of age	Jordan	LMIC	Transhumant and sedentary systems	Al-Khaza'leh *et al*., 2020
Early pre-weaning mortality	Deaths of young stock between 1-3 months of age	Ethiopia	LMIC	Mixed crop-livestock, pastoral/ agropastoral, & market-oriented urban/peri-urban dairy production	Fentie *et al*., 2016
Overall crude mortality rate	Number of deaths/Average number of lambs from birth to weaning age (3 months) during a one-year period	Jordan	LMIC	Transhumant and sedentary systems	Al-Khaza'leh *et al*., 2020
Post-neonatal mortality	Mortalities in the first 5-12 weeks of life	Turkey	LMIC		Gokce *et al*., 2013
Mean annual birth- to-weaning young stock mortality	Number of deaths of young stock from birth to weaning (up to 6 months for sheep) over one year/ Number of live births within a particular year	Ethiopia	LMIC	Mixed crop-livestock, pastoral/ agropastoral, & market-oriented urban/peri-urban dairy production	Fentie *et al*., 2016
Late pre-weaning mortality	Death of young stock between 3-6 months of age	Ethiopia	LMIC	Mixed crop-livestock, pastoral/ agropastoral, & market-oriented urban/peri-urban dairy production	Fentie *et al*., 2016
Post-weaning mortality	Death between 3-6 months of age/Total number of lambs born	India	LMIC	Semi-intensive and intensive systems on a research station	Mandal *et al*., 2007
Young stock mortality	Number of lambs that died aged 12 months or younger in one calendar year/Total number of kids born in the same calendar year	Afghanistan	LMIC	Not specified	Bartels *et al*. (2017)
Young animal mortality	Number of sheep <12 months that died during the observation period/ number of young animals bon in the same period	Afghanistan	LMIC	Village production systems	Schreuder *et al*., 1996
Lamb mortality	Number of deaths between birth and 12 months of age/Total number of lambs born	Ghana	LMIC	Traditional/semi-intensive	Turkson, 2003
Lamb survival	Number of lambs weaned per 100 lambs born dead or alive	Mexico	LMIC	Intensively reared lambs	Mellado *et al*., 2016
Annual adult mortality	Number of adult (>12 months old or mated) sheep that died (including emergency slaughter) during a one-year observation period/ number of adult animals present at the start of the year	Afghanistan	LMIC	Village production systems	Schreuder *et al*., 1996

^a^ As classified by The World Bank Group: LMIC = low- and middle-income country; HIC = high-income country

There was a dearth of articles reporting on mortality rates for goats. Only 11 articles were identified, from eight LMICs. These articles reported on 17 indicators ( Table 7). Perinatal, neonatal and pre-weaning mortality rate definitions were reported by three authors each. Two of the three authors defined perinatal mortality as death within the first 48 hours of birth, while two authors also defined perinatal mortality as that occurring between 48 hours and one month of age. All three authors reporting on pre-weaning mortality defined this as mortality occurring up to three months of age, although two authors measured this from birth, while one author measured this from one month of age.

**Table 7.** Definitions for goat mortality indicators reported in peer-reviewed literature.

Indicator	Definition/Rationale	Country	Income level ^a^	System (if available)	Reference
Early mortality	Mortality during first 48 hours	Israel	HIC	Semi-extensive	Rattner *et al*., 1994
Perinatal mortality	Mortalities within 48 hours of birth	Jordan	LMIC	Transhumant and sedentary systems	Al-Khaza'leh *et al*., 2020
	Death of live-born animals within 48 hours of life	Ethiopia	LMIC	Mixed crop-livestock, pastoral/ agropastoral, & market-oriented urban/ peri-urban dairy production	Fentie *et al*., 2020
	Total losses caused by abortions, stillbirths, and neonatal mortality (to 14 days of age) in one breeding season/Sum of aborted, stillborn and kids born alive	Jordan	LMIC	Extensively managed	Aldomy *et al*., 2009
Neonatal mortality	Number of mortalities between birth and 14 days of age/Total number of kids born alive	Jordan	LMIC	Extensively managed flocks	Aldomy *et al*., 2009
	Mortality between 48 hours to 1 month of age	Jordan	LMIC	Transhumant and sedentary systems	Al-Khaza'leh *et al*., 2020
	Deaths of newborn animals between 48 hours and 1 month of age	Ethiopia	LMIC	Mixed crop-livestock, pastoral/ agropastoral, & market-oriented urban/ peri-urban dairy production	Fentie *et al*., 2020
Pre-weaning mortality	Mortality between 48 hours and 70 days	Israel	HIC	Semi-extensive	Rattner *et al*., 1994
Overall kid mortality rate	Abortion rate + pre-weaning (<3mo) mortality rate	Sudan	LMIC	Traditional pastoralism	El-Hassan El-Abid & Nikhaila, 2009
	Number of deaths/Average number of kids from birth to weaning age (3 months) during one-year period. i.e. half of death was subtracted from live births as no other losses were encountered before weaning.	Jordan	LMIC	Transhumant and sedentary systems	Al-Khaza'leh *et al*., 2020
	Number of deaths in kids younger than six months/ Total number of kids born	Mali	LMIC	Traditional system	Ba *et al*., 1996
	Number of deaths between birth and 12 months of age/Total number of kids born	Ghana	LMIC	Traditional/semi-intensive	Turkson, 2003
Preweaning mortality	Number of deaths until 90 days of age/Total births	Ethiopia	LMIC	Smallholder farms using tethered feeding or pastoralist systems	Deribe *et al*., 2014
	Mortality from birth until weaning at 3 months of age	Sudan	LMIC	Traditional pastoralism	El-Hassan El-Abid & Nikhaila, 2009
	Mortality between 1-3 months	Jordan	LMIC	Transhumant and sedentary systems	Al-Khaza'leh *et al*., 2020
Early pre-weaning mortality	Deaths of young stock between 1-3 months of age	Ethiopia	LMIC	Mixed crop-livestock, pastoral/ agropastoral, & market-oriented urban/ peri-urban dairy production	Fentie *et al*., 2020
Mortality rate of suckling stock	Deaths in 0–6-month-old stock/ Animal- days*365*100	Malawi	LMIC	Crop/livestock smallholder farms	Chikagwa-Malunga & Banda, 2006
Mean annual birth-to- weaning young stock mortality	Number of deaths of young stock (up to 6 months for goats) in the study for one year/Number of live births within that particular study year	Ethiopia	LMIC	Mixed crop-livestock, pastoral/ agropastoral, & market-oriented urban/ peri-urban dairy production	Fentie *et al*., 2020
Post-weaning mortality	Mortality between 70 – 180 days	Israel	HIC	Semi-extensive	Rattner *et al*., 1994
Late pre-weaning mortality	Death of young stock between 3–6 months of age	Ethiopia	LMIC	Mixed crop-livestock, pastoral/ agropastoral, & market-oriented urban/ peri-urban dairy production	Fentie *et al*., 2020
Young stock mortality	Number of kids that died aged 12 months or younger in one calendar year/Total number of kids born in the same calendar year	Afghanistan	LMIC	Not specified	Bartels *et al*., (2017)
Young animal mortality	Number of goats <12 months that died during the observation period/ number of young animals bon in the same period	Afghanistan	LMIC	Village production systems	Schreuder *et al*., 1996
Mortality rate of rearing males	Deaths in 6-18-month-old males/ Animal- days*365*100	Malawi	LMIC	Crop/livestock smallholder farms	Chikagwa-Malunga & Banda, 2006
Mortality rate of rearing and breeding females	Deaths in females aged 6 months and older/ Animal-days*365*100	Malawi	LMIC	Crop/livestock smallholder farms	Chikagwa-Malunga & Banda, 2006
Annual adult mortality	Number of adult (>12 months old or mated) goats that died (including emergency slaughter) during a one-year observation period/ number of adult animals present at the start of the year	Afghanistan	LMIC	Village production systems	Schreuder *et al*., 1996
Mortality rate of breeding males	Deaths in >18 month-old males/ Animal- days*365*100	Malawi	LMIC	Crop/livestock smallholder farms	Chikagwa-Malunga & Banda, 2006

^a^ As classified by The World Bank Group: LMIC = low- and middle-income country; HIC = high-income country All studies are from low- and -middle income countries practicing mainly traditional forms of production.

A small number of studies arbitrarily divided the pre-weaning period into early and late stages of weaning, which differed greatly between authors. Papers that monitored mortality to weaning but did not specify when weaning typically occurred were excluded from the study. In the review article by Peeler & Wanyangu (1998), the authors collated the weaning ages of lambs and kids reported in grey literature from Kenya. Across 12 studies, weaning age ranged between 120 – 224 days, with an average of 152 days and a standard deviation of 30 days, showing significant variation within weaning ages of production systems used in one country.

Few studies reported mortality rates specific to adult animals - most studies reporting adult mortality rates used crude flock or herd mortality risk, rather than an age-based indicator. The exception to this is in dairy cattle, where a small number of studies reported mortality by parity in dairy cattle ( Traore & Wilson, 1988; Upadhyay *et al*., 2014).

Across all indicators for cattle, there was no more consistency in definitions used within HIC/LMIC groupings than between the two income categories. The small number or absence of studies from HICs for sheep and goats precluded comparisons between different production income groups. For all species, age-defined indicators concentrated on the first few months of life.

### Age distribution of mortality

While many articles discussing livestock mortality state that mortality rates are highest in younger age categories, few articles reported the distribution of mortality by age, particularly for animals older than one year. In cattle, 15 articles ( Table 8) presented detailed tables of mortality by age, allowing crude comparison between studies. However, for sheep and goats, this information is scarce and spread across varying timeliness, making direct comparison difficult.

**Table 8.** Characteristics of cattle studies included in age distribution of mortality graphing.

Reference	Country	Country income categorya	Breed	Production system characteristics	Study selection criteria	Total number of animals in study
Achard & Chanono, 1997	Niger	LMIC	Azaouak Zebu	Pasture-based breeding station	Institution records bounded by study dates	1,646
Bunter *et al*., 2014	Australia	HIC	Brahman and Tropical Composite	Extensively managed beef stations	Institution records bounded by study dates	9,296
Debnath *et al*., 1990	Bangladesh	LMIC	Mostly Bos Indicus breeds	Not specified	Institution records bounded by study dates	15,840
Debnath *et al*., 1995	Bangladesh	LMIC	Multiple, although Bos Indicus and Bos Taurus crossbreeds most common	Intensive dairy breeding station	Institution records bounded by study dates	8,623
Fuerst-Waltl & Fuerst, 2010	Austria	HIC	Holstein	Not specified; likely a mix of production systems as data from the Danish Cattle Database	Institution records bounded by study dates	86,249
Ganaba *et al*., 2002	Burkina Faso	LMIC	Zebu, Baoule and their crosses	Transhumant and sedentary mixed crop-livestock	Convenience sample	901
Gulliksen *et al*., 2009	Norway	HIC	Multiple dairy breeds	Not specified	Institution records bounded by study dates	289,038
Kudi *et al*., 1998	Nigeria	LMIC	Not specified	Traditional agropastoral	Convenience sample	277
Motus *et al*., 2018	Estonia	HIC	Mixed beef breeds	Extensive pasture-based	Institution records bounded by study dates	21,075
Prasad *et al*., 2004	India	LMIC	Sahiwal Zebu and Tharparkar Zebu and their crosses with Brown Swiss and Holstein Friesians	Organised dairy herd reared at a research institute	Institution records bounded by study dates	1,115
Pritchard *et al*., 2013	The UK	HIC	Holstein-Friesian	Not specified	Institution records bounded by study dates	112,163
Ring *et al*., 2018	Ireland	HIC	Multiple dairy breeds	Not specified; likely a mix of production systems as data from the Irish Cattle Breeding Federation database	Institution records bounded by study dates	11,256,112
	Ireland	HIC	Multiple beef breeds	Not specified; likely a mix of production systems as data from the Irish Cattle Breeding Federation database	Institution records bounded by study dates	9,839,949
Svensson *et al*., 2006	Sweden	HIC	Multiple dairy breeds	Intensive production	Institution records bounded by study dates	8,962
Upadhyay *et al*., 2017	India	LMIC	Sahiwal	Intensive dairy production at research institute	Institution records bounded by study dates	914
Wymann *et al*., 2006	Mali	LMIC	Zebu, N’Dama, and crossbreeds with European breeds	Per-urban traditional agro- pastoralism, modern, and station- managed systems	Convenience sample	784

^a^ As classified by The World Bank Group: LMIC = low- and middle-income country; HIC = high-income country.

**Figure 2.**
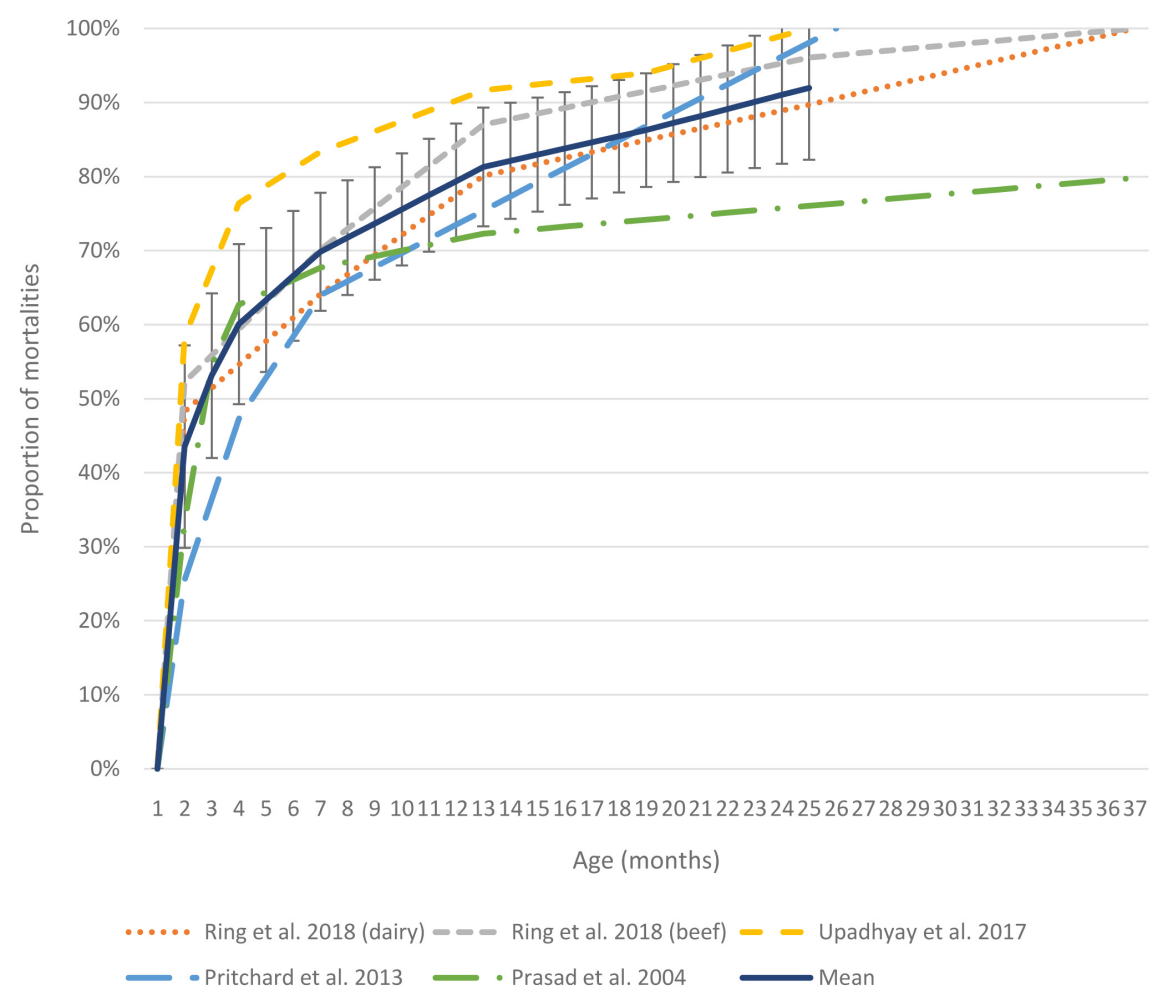
Cumulative mortality in cattle from birth to three years of age. Four included studies presented data on mortality distribution by age past 12 months of age. The black line shows the mean cumulative mortality with standard deviation bars.

***Mortality in adult cattle.*** Only four articles detailed age at mortality to at least three years of age. Data from these articles are presented in Figure 2. It is evident that mortality rises steeply in the first few months of life and begins to taper off by 12 months of age.

In addition to these studies reporting exact numbers, several papers presented cumulative mortality (or proportion of survival) over time graphically. In Mali, Traore & Wilson (1988) showed that the proportion of cattle surviving dropped most steeply within the first three months of life, then declined at a fairly steady rate between 1–3 years, before becoming negligible between 3–4 years. Raboisson *et al.*, 2013 showed that in three different dairy cattle breeds in France, heifer survival rates dropped most rapidly within the first 200 days of age, continuing at moderate rates between 200–400 days, before reaching a lower, stable rate between 400–1400 days. Zhang *et al.* (2019) reported on mortalities and involuntary culling rates in dairy calves and replacement heifers in China to 60 months of age. Frequency of mortality was highest in the <3-month age group, dropped dramatically between 3–6 months, then continued to drop until 60 months of age.

**Figure 3.**
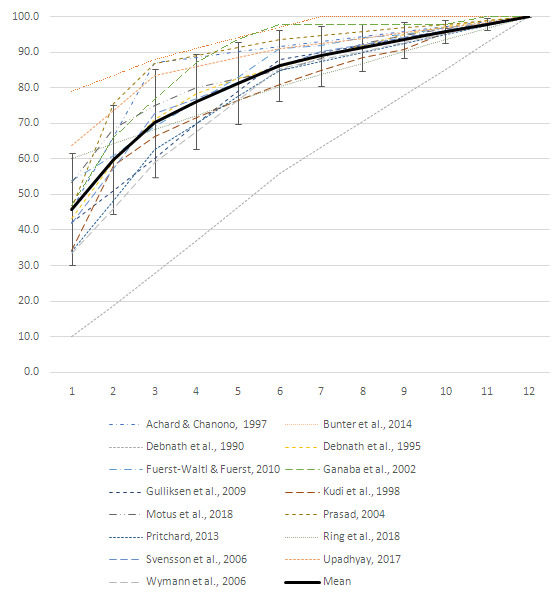
Cumulative mortality in calves between 0 - 12 months of age. This graph shows includes 15 studies reporting mortality distribution by age for at least the 12 months of age. The black line shows the mean cumulative mortality with standard deviation bars. On average, 86% of mortalities in the first 12 months had already occurred by six months of age.

***Mortality in young cattle.*** For cattle in the first 12–15 months of life, a total of 11 articles reported detailed mortality incidence risk over time. Study sizes, age groups for which data are presented and proportions of total mortality are included in Table 9. As demonstrated in the table, there is inconsistency in the age groupings used. Three studies reported mortality risk for each month, however, one study reported mortality for each 28-day period. Other studies reported mortalities for arbitrarily-determined age groups. To be able to compare and present this data graphically, figures were either averaged or consolidated to give monthly values, and this cumulative mortality risk for the first year of life is presented in Figure 3. For studies reporting mortality risk monthly, cumulative mortality rises sharply in the first 2–3 months. In all but one study, 80% of mortalities that occur in the first year have occurred by six months of age.

**Table 9.** Risk of bias of studies included in the age distribution of mortality study, using the Risk of bias tool ( Hoy *et al.,* 2012).

Study	1. Was the study’s target population a close representative of the national population in relation to relevant variables?	2. Was the sampling frame a true or close representation of the target population?	3. Was some form of random selection used to sample the selection, or, was a census undertaken?	4. Was the likelihood of non-response bias minimal?	5. Were data collected directly from the subjects?	6. Was an acceptable case definition used in the study?	7. Was the study instrument that measured the parameter of interest shown to have reliability and validity (if necessary)?	8. Was the same mode of data collection used for all subjects?	9. Was the length of the shortest prevalence period for the parameter of interest appropriate?	10. Were the numerator(s) and denominator(s) for the parameter of interest appropriate?	11. Summary item on the overall risk of study bias ^
Achard & Chanono, 1997	Yes	Yes	N/A	Yes	Yes	Yes	Yes	Yes	Yes	Yes	Low
Bunter *et al*., 2014	Yes	Yes	N/A	Yes	Yes	Yes	Yes	Yes	Yes	Yes	Low
Debnath *et al*., 1990	Yes	Yes	N/A	Yes	Yes	Yes	Yes	Yes	Yes	Yes	Low
Debnath *et al*., 1995	Yes	Yes	N/A	Yes	Yes	Yes	Yes	Yes	Yes	Yes	Low
Fuerst-Waltl & Fuerst, 2010	Yes	Yes	N/A	Yes	Yes	Yes	Yes	Yes	Yes	Yes	Low
Ganaba *et al*., 2002	Yes	No	No	Yes	Yes	Yes	Yes	Yes	Yes	Yes	Moderate
Gulliksen *et al*., 2009	Yes	Yes	N/A	Yes	Yes	Yes	Yes	Yes	Yes	Yes	Low
Kudi *et al*., 1998	Yes	No	No	Yes	Yes	Yes	Yes	Yes	Yes	Yes	Moderate
Motus *et al*., 2018	Yes	Yes	N/A	Yes	Yes	Yes	Yes	Yes	Yes	Yes	Low
Prasad *et al*., 2004	Yes	Yes	N/A	Yes	Yes	Yes	Yes	Yes	Yes	Yes	Low
Pritchard *et al*., 2013	Yes	Yes	N/A	Yes	Yes	Yes	Yes	Yes	Yes	Yes	Low
Ring *et al*., 2018	Yes	Yes	N/A	Yes	Yes	Yes	Yes	Yes	Yes	Yes	Low
Svensson *et al*., 2006	Yes	Yes	N/A	Yes	Yes	Yes	Yes	Yes	Yes	Yes	Low
Upadhyay *et al*., 2017	Yes	Yes	N/A	Yes	Yes	Yes	Yes	Yes	Yes	Yes	Low
Wymann *et al*., 2006	Yes	No	No	Yes	Yes	Yes	Yes	Yes	Yes	Yes	Moderate

Yes = low risk; No = high risk. ^ **Low risk:** Further research is very unlikely to change our confidence in the estimate. **Moderate risk:** Further research is likely to have an important impact on our confidence in the estimate and may change the estimate. **High risk**: Further research is very likely to have an important impact on our confidence in the estimate and is likely to change the estimate.

Again, these observations are supported by many of the other studies that either did not report exact numbers over time or that presented data over a shorter period of time. Findings reported by authors included those where the largest proportion of deaths in cattle occurred intrapartum ( Mock *et al*., 2020), in the first 48 hours ( Busato *et al*., 1997; Raboisson *et al*., 2013), the first week ( Gardner *et al*., 1990), first one- ( Menzies *et al*., 1996), two- ( Santman-Berends *et al*., 2019), or three- ( Hyde *et al*., 2020) months of life.

For both extensively-managed beef cow-calf herds and large-scale, intensive dairy cattle production in Estonia, Motus *et al.* (2017) and Reimus *et al.* (2017) found that mortality rates were highest in female and male calves 0–3 months of age. For beef cattle, mortality rates dropped markedly after 3 months until 18 months of age in females, when they began to rise again, while mortality rates in male calves was more variable. In dairy cattle, for both sexes, mortality rates remained moderately high between 3–5 months, before dropping at 6 months of age. Data available for older age groups in female cattle show that mortality rates start to rise again at 24 months. Similarly, Selvan *et al.* (2019) reported mortality risk for two Zebu breeds and Zebu crossbreed calves under 6 months of age in India from longitudinal data from a research station. For the two Zebu breeds (Sahiwal and Tharparkar), mortality risk was highest in calves aged 0–1 month, whereas mortality risk was comparatively high for calves 0–1 month and 1–3 months of age for the crossbred calves. Norberg *et al*. (2013) studied mortality in Danish Jersey heifer calves aged 1–180 days and found that the risk of mortality was highest between days 1–14, moderate between days 15–30, and continued to decrease until 180 days of age. Slavik *et al*. (2009) found that in beef cattle herds in the Czech Republic, 62% of mortalities within the first six months occurred in the first week, with a further 28% occurring between one and four weeks of age.

In smallholder mixed crop-livestock systems in Kenya, Thumbi *et al*. (2013) found three periods where risk of cattle mortality was higher: the neonatal period, immediately after birth; between 150–190 days, when maternal antibody levels are waning; and towards one year of age, when calves are weaned. Knopf *et al*. (2014) reported that all deaths occurred in the first 210 days, while Pannwitz (2015) recorded the highest mortality rates in calves less than six months of age, then a decreased mortality rate between 6–24 months, and an uptick after 2 years of age.

In contrast to these findings, two authors, Debnath *et al*. (1990) ( Figure 3) and Gitau *et al*. (1994) reported that no strong age patterns were seen in calf mortalities, while Seppa-Lassila *et al*. (2016) found that mean mortality risk in large-scale Finnish dairy cattle was 5.2 ± 2.3% in calves <7 days, while the mortality risk in calves 7–180 days was 5.7 ± 6.2%.

***Risk of bias of included studies.*** For the 15 studies that published data sets showing age distribution of mortality in cattle, risk of bias was assessed using a tool refined by Hoy *et al.* (2012) ( Table 10). All seven studies from HICs and five of the studies from LMICs utilised data from national registries, or research/breeding institutes, therefore random participant selection and non-response bias was unable to be assessed. It is possible that research or breeding station data may not be a true representation of realities in the field. Although some herds were managed as per the local normal, some stations had much more intensive management than would be found in surrounding areas. For the remaining three LMIC studies, these were conducted in the field using convenience sampling, based on farms being in accessible locations, being the site of previous studies, and willingness to participate by farmers. Convenience sampling may select participants who have a greater interest in the health and welfare of their cattle and therefore may employ better management practices, or who have better access to veterinary advice or treatments. Overall, the risk of bias in studies originating from HICs was assessed to be low, and moderate - though difficult to avoid due to constraints on how research can be undertaken - in studies originating from LMICs. Other types of bias, such as reporting bias, were not reported by studies and could not be assessed.

**Table 10.** Proportion of mortalities in calves under 15 months, by age group reported by respective authors.

Reference	Days	0-2	2-7	7-14		14-28														
	Months						2	3	4	5	6	7	8	9	10	11	12	13	14	15
Achard & Chanono, 1997		36% (51)			51% (72)				4% (6)			8% (11)								
Bunter *et al*., 2014		66% (589)		13% (116)			9% (80)		12% (107)				0%							
Debnath *et al*., 1990		9.9% (48)					17.8% (86)		28.1% (136)			44.2% (214)								
Debnath *et al*., 1995		24.2% (277)		9.8% (113)	8% (92)	15% (172)	12% (141)	7% (84)	5% (52)	3% (36)	3% (32)	3% (30)	3% (35)	3% (33)	2% (20)	1% (17)	1% (12)			
Fuerst-Waltl & Fuerst, 2010a			54% (1596)				37% (1095)					9% (278)								
Ganaba *et al*., 2002		21% (10)	26% (12)				19% (9)	11% (5)	11% (5)	6% (3)	4% (2)	0% (0)	0% (0)	0% (0)	0% (0)	2% (1)	0% (0)			
Gulliksen *et al*., 2009		24% (2515)		18% (1937)			47% (4971)					12% (1243)								
Kudi *et al*., 1998		34% (45)					24% (31)	8% (11)	5% (7)	5% (6)	5% (6)	4% (5)	4% (5)	2% (3)	5% (6)	2% (3)	2% (3)			
Motus *et al*., 2018		2% (12)	15% (116)	21% (161)		16% (120)	15% (115)	7% (50)	5% (39)	3% (20)	2% (17)	3% (26)	4% (32)	2% (17)	2% (16)	1% (9)	2% (16)			
Svensson *et al*., 2006		40% (154)					29% (113)		17% (65)				15% (57)							
Wymann *et al*., 2006		23% (21)			9% (8)		24% (22)		25% (23)			20% (19)								

The mortality risk is presented as a percentage (number of deaths over the number of animals recorded in this age group over the defined study period), with the number of mortalities included in brackets. Cumulative mortality incidence may give a total of just under or just over 100% due to rounding. This table shows the variation between studies in age groups selected for study. ^a^ Deaths occurring between birth and 48 hours were considered as stillbirths by the authors and are not included in the study.

***Mortality by age in small ruminants.*** For both sheep and goats, the variation in the time periods covered by different studies was too great to allow direct comparisons. In sheep, two papers noted that stillbirth and perinatal mortality rates were higher than at any age ( Holmoy & Waage, 2015); Voigt *et al.*, 2019), while other authors report the largest proportion of mortalities occurred within 24 hours of birth ( Binns *et al*., 2002), or within first week ( Gokce & Erdogan, 2009; Gokce *et al*., 2013; Khan *et al*., 2006). Holmoy *et al*. (2017) reported that 80% of neonatal lamb mortalities occurred within the first two days of life. Sallam (2019) found that average mortality risk for Barki sheep reared at a research station in Egypt was 4.6% for lambs 0–3 days of age, 5.4% for lambs 4–60 days of age, and 2.2% for lambs 61–90 days of age. Turkson (2003) found that for lambs from birth to 12 months of age, 75.6% of the mortalities occurred between 0–3 months, compared to 24.4% occurring between 4–12 months of age. In India, Mandal *et al*. (2007) reported that mortalities of Muzaffarnagari sheep reared in a research institute were highest in lambs under six months of age. Mortality risk for lambs from 0–3 months of age was 7.5% (with 64% of deaths occurring within 15 days of birth), decreasing to 2.7% from 3–6 months of age, 1.6% from 6–9 months of age, and 1.2% from 9–12 months of age ( Mandal *et al*., 2007). In Jordan, Abdelqader *et al*. (2017) found that 63.5% of pre-weaning (from birth to 60 days) lamb mortalities occurred within the first seven days of life. In semi-migratory and village sheep production systems in Iran, Vatankhah & Talebi (2009) found that mortality risk was 6.14%, 12.76%, 3.36% and 0.69% for lambs under 3 months of age, between 3 and 6 months, 6 and 9 months, and 9 and 12 months of age, respectively.

For goats, El-Hassan El-Abid & Nikhaila (2009) observed that 21.2% of pregnancies resulted in abortion, while de Medeiros *et al*. (2005) reported the highest proportion of mortalities occurring within the first month. Traore & Wilson (1988) reported that 35% of all kids died before five months of age in Mali. For both sheep and goats, Tifashe *et al*. (2017) observed that lambs and kids had higher mortality rates than “young stock” or “adults”, however, the specific age range covered by these terms were not defined. Kumar *et al*. (2016) reported that risk of mortality was highest in kids aged 0–3 months (34.6%), >3–6 months (25.42%), and >6 months (19.78%). Turkson (2003) found that for mortalities occurring in kids between birth and 12 months of age, 80.2% occurred between 0–3 months, with the remaining 19/8% occurring between 4–12 months. In India, Thiruvenkadan & Karunanithi (2007) longitudinal data from research station records showed that mortality risk was higher in kids from birth to 12 months of age compared to adults older than 12 months. In this cohort, mortality risk was highest around the time of weaning at three months of age.

In Myanmar, a study on village sheep and goats production found that mortality rates were much higher in young animals aged less than 12 months compared to older animals above 12 months (3.0 deaths/100 animals/month and 0.28 deaths/100 animals/month respectively) ( Hanks *et al*., 2018).

Ramachandran *et al*. (2006) reported on longitudinal data on an experimental crossbred dairy goat herd maintained at a research institute. For goats monitored until 78 months of age (6.5 years), 59% of all mortalities occurred in the first 3 months of age, and 72% of mortalities had occurred by 6 months of age. After 6 months of age, mortality risk dropped markedly, and remained low until the goats were >78 months of age.

## Discussion

### Inconsistencies in definitions

The inconsistency between definitions of livestock mortality indicators suggests that mortality indicators are not used consistently enough across the industry to be standardised. This is likely due to the predominance of performance indicators based on productivity in HICs, such as daily weight gain, feed conversion ratios, carcass or milk yield or egg production; the irregularity of monitoring in LMICs; and the difficulties in standardising these indicators so that they are applicable across different geographical regions and production systems. However, livestock mortality indicators may become more widely used globally - in HICs due to increasing consumer concerns about animal welfare and the potential utility of mortality indicators in this domain, and in dairy cattle, due to concern about the rising trend in mortality rates seen over the last few decades ( Compton *et al*., 2017; de Vries *et al*., 2014; Thomsen & Houe, 2018), and in LMICs due to the inclusion of “Number of animal deaths” in FAO’s recommended minimum set of core data within the Global Strategy to improve Agriculture and Rural Statistics ( FAO, 2018). In this case, a concerted effort should be made to overcome the difficulties in standardising livestock mortality indicators, as this will facilitate comparisons over time. Several sources of inconsistency that need to be addressed have been identified in this review.

First is the use of mortality risk versus mortality rate. Santman-Berends *et al*. (2019) compared practical aspects and suitability of mortality rates, where calf days-at-risk was used as the denominator, to mortality risk, where the total number of animals at a specified point in time is used. These authors found that although mortality rate is more accurate, mortality risk was a preferred method of measuring mortality from the scientific, comprehensibility and utilitarian points of view. This finding was supported by the high proportion of studies that reported mortality risk rather than rate. In many cases, method of data capture may not have given authors the degree of precision required to calculate mortality rate. To improve ease of comparison between studies, it may be helpful for studies with access to more detailed data to report both mortality rate and mortality risk, to facilitate comparison with studies with access to less precise data that report mortality risk.

Secondly, based on the studies reviewed here, stakeholders have a greater interest in mortality in young animals. As perinatal and neonatal mortality rates are commonly reported, species-specific definitions for these indicators using age ranges that are appropriate for use across different production systems should be set. For both cattle and goats, more papers defined perinatal mortality as occurring within the first 48 hours of life than other time periods, although variation exists as to whether abortions or stillbirths are included. Due to potential inconsistencies in the detection of abortion or determination of foetal age at abortion under field conditions, the authors propose that perinatal mortality risk be defined for cattle, sheep and goats as animals that are stillborn or die within 48 hours of birth over the total number of still- and live-born animals. For cattle, sheep and goats, most authors defined the upper age limit of neonatal mortality as one month of age. The authors propose that neonatal mortality risk for all three species include deaths occurring from three to 30 days of age over the total number of animals alive at three days.

The literature search for the term “pre-weaning mortality” showed that this is a more commonly used indicator for pig production systems than for ruminants. Conceptually, pre-weaning mortality would be a useful indicator in ruminants as it encompasses the age groups with highest risks of mortality. However, due to the high variability in age at weaning between production systems and species, an indicator based on age may be more universally appropriate. For cattle, studies that report pre-weaning mortality rates should, at minimum, specify the age at weaning for the setting of the study. For sheep and goats, reviewed papers seem to concur that the pre-weaning period should extend to 90 days of age. The authors suggest that pre-weaning mortality risk for sheep and goats be defined as the number of liveborn animals that die between birth and 90 days of age over the total number of liveborn animals.

Lastly, for animals past the weaning stage, the age groups for which mortality rates are reported could be standardised. At present, study authors arbitrarily decide on age groupings for reporting or further analysis. In a review attempting to compare magnitude of calf loss across cattle stations in Northern Australia, Chang *et al*. (2020) identified 42 studies that reported mortality over 13 different time periods. This variation in timelines limited the usefulness of the data, precluding meta-analysis and allowing only summary statistics to be generated. Although the length of studies may vary, if all studies reported on mortality rates for a consistent set of age ranges, this would aid comparisons across data sets and meta-analyses for more powerful results.

### Age at mortality

This review found high agreement between studies on the age groups with the highest incidences of mortality in cattle, sheep and goats, although most evidence was available for cattle. Mortality rates were clearly higher in young animals within the first few months of life, and by six months of age, a large proportion of mortalities in herds and flocks had already occurred. This appears to be a common finding regardless of geographic location or production system and is likely why most studies concentrated on reporting mortality rates for younger animals rather than adults.

While it is commonly reported anecdotally in the literature that the perinatal period or first week of life is the most dangerous period for small ruminants, studies from LMICs also reported higher risk of mortality around the time of weaning, which extends the period where higher mortality risk is observed to 6 months of age. Given the findings in this review, young stock mortality risk, where the number of animals dying within six months of birth over the number of live-born animals could be used as an indicator to cover this vital period. Considering the ability for human infant mortality rates to reflect general population health ( Reidpath & Allotey 2003), the relationship between young stock mortality risk and overall herd or flock health could be an area for further analysis.

### Limitations of the study

There are several limitations to this review. Firstly, the study was limited to one citation database and limited use of Google Scholar. Web of Science was chosen for the breadth of journals indexed within this database and their relevance to livestock science. A large number of search results were generated, however, potentially more articles could have been recovered using a second citation database. Secondly, the study criteria restricting inclusion to peer-reviewed journal articles to ensure a high quality of studies may have limited the amount of data included from LMICs, where there may be language or financial barriers to publishing in peer-reviewed journals, and where operational research from governmental or non-governmental organisations may only be published in the grey literature. Finally, an inadequate number of studies reporting sufficient age distribution of mortality data were identified to conduct a meta-analysis or to disaggregate findings by production system or location.

### Practical considerations of mortality rate as an indicator

***Availability of data.*** While close to 50% of the cattle studies included in this review originated from HICs, sheep studies more commonly originated form LMICs, and all included goat studies were from LMICs. In HICs, farmers routinely collect data for the purposes of animal identification, registration, and performance monitoring. Due to the availability of these registries, national-level trends are relatively easy to map. Evidence from LMICs is much scarcer, with studies on mortality usually reporting on small-scale retrospective surveys or using data from institutional research or breeding stations. Lesnoff (2009) found that retrospective survey methods for estimating mortality rates over a 12-month period were fairly reliable for cattle and acceptably reliable for small ruminants, however, care is still required in interpreting survey results due to the large degree of variation within and between years. Season, large shocks, and innovations targeting herd productivity can lead to marked variation, which can affect survey results depending on when they are conducted, and the period(s) covered. Variability was higher for small ruminants than cattle, due to higher reproduction and mortality rates. To limit bias from variability, Lesnoff (2009) recommends that whole herd monitoring over several years, rather than 12-month retrospective surveys, should be employed for data collection and analysis where possible. Currently, this is difficult to achieve in LMICs, where monitoring tools are largely unavailable or unused, national livestock registries do not exist, and the reporting systems and investigation of mortality events are still in nascent form.

***Data quality.*** Countries that maintain national databases for livestock registration appear to have robust and complete records including mortality events. However, the grace period for registration of new animals or the requirement for ear-tagging or other forms of identification to be completed before registration mean that in some cases, mortality within the first few days of life may be underreported ( Motus *et al*., 2018; Ortiz-Pelaez *et al*., 2008; Raboisson *et al*., 2013). A large proportion of young stock mortalities occur within the first seven days of life, particularly in small ruminants, so such unrecorded omissions may be significant.

Data quality may also be an issue in countries that rely on retrospective surveys. Accurate reporting of mortality can be influenced by the skill of the interviewer, and the farmer’s ability to accurately recall mortality events ( Lesnoff, 2009). Of the studies reviewed, Debnath *et al*. (1990) were the only authors to discuss the recall accuracy of farmers. These authors reported that farmers were able to reliably recall livestock mortality events, however, the exact ages of animals that died were more difficult to pinpoint. Farmers were more confident in reporting the age group of the animal. In addition to problems of recall, interviewers may encounter reluctance to report mortality, particularly in areas where disease surveillance has previously led to uncompensated control measures to stamp out disease ( Gilbert, 2012; Otte *et al*., 2004).

***Cause of mortality.*** In the reviewed papers, it was rare for mortality rates in themselves to be the sole focus of the study: identifying actual or potential causes of mortality or using modelling to identify risk factors was also an important component. This denotes a major weakness in the use of mortality rates to monitor trends in livestock health; mortality rates only indicate the magnitude of the problem. To be able to make improvements, more information is required for interventions to be able to target to the underlying problems. In areas and/or age groups where the causes for mortality remain consistent over time, trends in mortality rate may be useful to track progress or decline, and to monitor for outbreaks. However, both causes and risk factors may vary widely over geographical and temporal scales. In some regions of the world where livestock mortality rates are consistently high, livestock keepers may be exposed to a variety of shocks year on year, including those related to climate or extreme weather events, political and social stability, and human or animal disease epidemics. In the FAO guidelines for estimating livestock production in LMICs, to monitor herd dynamics and animal health, it is recommended to measure the “number of deaths or disappearance per livestock species and by cause” ^1^ rather than just mortality rate ( FAO, 2018).

Several of the papers included in this review which presented data spanning multiple years noted that mortality varied greatly from year to year. It was postulated that this was due to environmental stresses such as feed or water shortage, or from disease outbreaks. For livestock mortality data to be able to be useful in a timely manner, long term data needs to be collected and accessible. Causes of fluctuations in mortality rates need to be noted, and a “baseline” mortality rate for that region established from the years without external events. This is a similar concept to the “excess deaths” indicator currently being monitored for human mortalities during the COVID-19 pandemic. For livestock, where mortalities follow a seasonal pattern, baseline mortality rates should be established for each season so that if mortality rates rise above baseline at any time through the year, rapid investigation and action is achievable.

## Conclusion

This systematic review finds that although mortality indicators are used to monitor ruminant production systems in both HICs and LMICs, there is a lack of consistency between age groups monitored, time periods covered, and denominators used. It is likely that mortality rate will continue to be used in both HICs and LMICs for the purposes of monitoring animal health or welfare, and comparisons between studies and over time would be aided by the use of standard definitions. The highest proportion of mortalities in cattle, sheep and goats is reported to occur within the first six months of life; therefore, this would be a useful age group over which to measure young stock mortality risk.

However, in itself, mortality rate is an incomplete indicator due to the high level of variability in causes and risk factors. To better understand variations in mortality rate between years, or to target preventative actions, the monitoring of mortality rate should be supplemented with qualitative or quantitative data on likely causes of livestock mortality where possible.

Globally, for the livestock community to increase the utility of data generated and accelerate progress towards improved animal health and welfare, the authors recommend the following actions:

- To improve the interoperability of mortality indicators used for research and monitoring and evaluation, including:
  ◦ Improving accuracy in the use of the terms “risk” and “rate”. As mortality risk is more widely used and requires less data to calculate, authors should aim to always report mortality risk, supplementing with mortality rate where possible.
  ◦ Standardising common indicators such as:
    ▪ Perinatal mortality risk, which could be defined in cattle, sheep and goats as including stillbirths and deaths until 48 hours after birth;
    ▪ Neonatal mortality risk, which could be defined in cattle, sheep and goats as including deaths occurring between three and 30 days of age;
    ▪ Pre-weaning mortality risk for sheep and goats as death of liveborn animals between birth and 90 days of age; and
    ▪ Young stock mortality risk in cattle, sheep and goats as death of liveborn animals between birth and six months of age.
  ◦ Selecting appropriate adult age groups for which all researchers can collect data for and report against.
- To support farmers in establishing herd monitoring practices and increasing investment in the creation of national livestock registries.
- To further investment in initiatives that support farmers in establishing and recording the underlying causes of livestock mortality.

Together, these actions will enable farmers to understand the trends and underlying factors causing livestock mortality, and enhance the interoperability and value of data generated from different livestock surveillance and research projects.

## Data availability

All data underlying the results are available as part of the article and no additional source data are required.

### Reporting guidelines

Harvard Dataverse: “Refining livestock mortality indicators: A systematic review PRISMA checklist”. https://doi.org/10.7910/DVN/JJIHJG ( Wong, 2021).

Data are available under the terms of the Creative Commons Zero "No rights reserved" data waiver (CC0 1.0 Public domain dedication).
