# Supplementary material for: Refining livestock mortality indicators: a systematic review
Source: Gates Open Res. 2021 Apr 19;5:75. [Version 1] doi: 10.12688/gatesopenres.13228.1 (PMC8358167; doi:10.12688/gatesopenres.13228.1)
Supplement: Peer review history

# Peer review: Reviewer response for version 1

**DOI:** 10.21956/gatesopenres.14456.r30999

**Published:** 2021-08-17

**Authors:** Julia Adriana Calderón Díaz, Martyna Lagoda

**Affiliations:** 1 Pig Development Department, Teagasc Animal and Grassland Research and Innovation Centre, Fermoy, Ireland

We would like to congratulate the authors in preparing a very comprehensive and well structured literature review. The article tries, for the first time, to harmonise definitions for mortality in cattle and small ruminants which could be used as a first step to develop and/or improve animal health surveillance programs mostly in low-middle income countries. Livestock mortality negatively affects the livelihoods of those involved in animal production, and addressing the issue identified in this review thus has the potential to improve monitoring, as well as the development of practical solutions to ameliorate increasing livestock mortality. The topic of this review is therefore of great value.

 The authors give a clear overview of the background information needed to understand the rationale for this systematic review, and also provide an easy to follow outline of the literature search technique used to conduct this review. Throughout the article, the authors point out to the inconsistency in the use of mortality indicator definitions in the existing literature and some of the main challenges to set standard definitions, and indicator variables across species, production systems and geographical areas.

 Lots of very useful information on the reviewed articles is concisely presented in the tables used. If we can recommend something in the article, that would be the information presented in the figures. We found the line patterns used to represent the information from various studies in Figure 3 quite difficult to distinguish. If possible, we would recommend that the authors try to make this figure clearer. In addition, the word “includes” within the caption of Figure 3 should be removed.

 Finally, based on the information deduced by the authors from the articles which they reviewed, the recommendations for future work and potential terms to be used when discussing livestock mortality seem very well formed and are likely to be of value to future authors.

Are the rationale for, and objectives of, the Systematic Review clearly stated?

Yes

Is the statistical analysis and its interpretation appropriate?

Yes

Are sufficient details of the methods and analysis provided to allow replication by others?

Yes

Are the conclusions drawn adequately supported by the results presented in the review?

Yes

Reviewer Expertise:

Livestock production; Animal welfare, Whole farm modelling; Stochastic bio-economic modelling; Bio-security

We confirm that we have read this submission and believe that we have an appropriate level of expertise to confirm that it is of an acceptable scientific standard.

# Peer review: Reviewer response for version 1

**DOI:** 10.21956/gatesopenres.14456.r30998

**Published:** 2021-08-11

**Authors:** Ishmael Festus Jaja

**Affiliations:** 1 Department of Livestock and Pasture Sciences, Risk and Vulnerability Science Centre, University of Fort Hare, Alice, South Africa

**General comments:**

 The manuscript “Refining livestock mortality indicators: a systematic review", presents a compelling quantitative description of livestock mortality indicators vis-a-vis age delineation. Based on its finding, the authors proposed using mortality risk to improve the interoperability of livestock mortality indicators. They further suggested the standardizing common indicators such as perinatal, preweaning, neonatal and young stock mortality risk. The manuscript is well written and easy to understand. I found a few typographical errors, and I also suggest the authors reading the draft repeatedly to correct such errors.

**Comments:**

- “An overview of the criteria used to identify literature relating to the two purposes of this review is presented in in Table 4” - Delete “in”, it was typed twice.
- The authors stated, “Given the scoping nature of this section of the review, studies were not individually assessed for bias” - My opinion of the paper, given the risk of bias and its non-assessment, the authors could better call this paper a scoping review.

Are the rationale for, and objectives of, the Systematic Review clearly stated?

Yes

Is the statistical analysis and its interpretation appropriate?

Partly

Are sufficient details of the methods and analysis provided to allow replication by others?

Yes

Are the conclusions drawn adequately supported by the results presented in the review?

Yes

Reviewer Expertise:

Ishmael Festus Jaja, is a veterinarian in the Department of Livestock and Pasture Science, University of Fort Hare, Alice, South Africa. He obtained his DVM from the University of Nigeria, Nsukka, MSc and PhD from the University of Fort Hare, South Africa. He specializes in Veterinary public health, food safety, especially foodborne pathogen, and antimicrobial resistance.

I confirm that I have read this submission and believe that I have an appropriate level of expertise to confirm that it is of an acceptable scientific standard.

# Peer review: Reviewer response for version 1

**DOI:** 10.21956/gatesopenres.14456.r30819

**Published:** 2021-07-28

**Authors:** Alasdair J.C. Cook

**Affiliations:** 1 Department of Veterinary Epidemiology and Public Health, School of Veterinary Medicine, University of Surrey, Guildford, UK

This review addresses an important issue with respect to assessment of the health and productivity of livestock - the use of mortality data. The authors conducted a formal literature review and identified 85 articles for data extraction. Most of these related to cattle. Useful tables are presented showing the range of definitions related to mortality by species and age group, emphasising the lack of consistency that is seen. Data were summarised to present useful cumulative mortality curves for cattle; the standard deviation bars are wide illustrating the great diversity in mortality within age groups and between countries. The authors recommend terminology related to mortality rate and risk is adhered to by authors and also stress the importance of defining the age groups to which such measures are applied. They also emphasise that a crude measure of mortality alone is of limited value and that cause-specific measures and/or additional qualitative or quantitative information should be provided.

 This is an excellent and authoritative article; it should be widely used as a reference by future authors concerned with reporting livestock mortality and also be useful as a guide to reviewers of such articles.

Are the rationale for, and objectives of, the Systematic Review clearly stated?

Yes

Is the statistical analysis and its interpretation appropriate?

Yes

Are sufficient details of the methods and analysis provided to allow replication by others?

Yes

Are the conclusions drawn adequately supported by the results presented in the review?

Yes

Reviewer Expertise:

I am a veterinary epidemiologist with research interests in infectious diseases and zoonoses, one health, the surveillance of livestock disease and in the health and productivity of livestock in production systems in developed countries and LMICs

I confirm that I have read this submission and believe that I have an appropriate level of expertise to confirm that it is of an acceptable scientific standard.
